# Guillain-Barré syndrome as the first presentation of human immunodeficiency virus infection

**DOI:** 10.1186/s12883-021-02350-1

**Published:** 2021-08-18

**Authors:** Mariana Lopes, Patrícia Marques, Bruno Silva, Gonçalo Cruz, José Eduardo Serra, Eugenia Ferreira, Helena Alves, José Saraiva da Cunha

**Affiliations:** 1 Department of Infectious Diseases, Centro Hospitalar e Universitário de Coimbra, Coimbra, Portugal; 2 Department of Neurology, Centro Hospitalar e Universitário de Coimbra, Coimbra, Portugal; 3 Department of Infectious Diseases, Centro Hospitalar e Universitário de Coimbra, Coimbra, Portugal

**Keywords:** Guillain-Barre Syndrome, HIV, Antiretroviral Therapy

## Abstract

**Aim:**

Antiretroviral therapy (ART) development has reduced the severity of neurological complications of the human immunodeficiency virus (HIV), but they remain prevalent and need prompt recognition. Acute inflammatory demyelinating polyneuropathy (AIDP) is a rare complication of human immunodeficiency virus (HIV) infection that may appear at any stage of the disease. In this case, AIDP represents a late presentation of HIV infection.

**Methods:**

Descriptive study. Patient data were collected from their medical records and by health assessment interviews.

**Results:**

We report a case of a 52-year-old male with acute lower limb weakness. Given the suggestive clinical presentation of AIDP and a positive HIV test, intravenous immunoglobulin (IVIG) was administered along with antiretroviral therapy. Progressive weakness to the upper limbs, autonomic dysfunction, and pain was observed. The second regimen of IVIG plus corticosteroids was administered. Muscle strength improved after three weeks.

**Conclusions:**

Screening for HIV in a patient with AIDP may provide a better outcome because of the early start of ART with good central nervous system penetration in HIV-infected patients.

## Background and aims

Guillain-Barre syndrome (GBS) is often designated as an acute inflammatory demyelinating polyneuropathy (AIDP) – the most common variant of the syndrome. It is defined by symmetric muscle weakness, loss of sensation, and loss of deep tendon reflexes [1].

It is typically considered a post-infection autoimmune disease, with more than 2/3 of the cases following a period of respiratory symptoms or gastroenteritis. An incidence rate of fewer than 2 cases per 100,000 is reported in Europe, and it is about twice as common in males [2, 3].

Clinical deficit peaks in 2–4 weeks, and the recovery period can last months or years, with 20% of patients developing long-term neurological sequelae [2]. Respiratory muscle weakness and autonomic dysfunction can develop during the course of the disease, and 4–15% of the cases may end in death [3].

After the human immunodeficiency virus (HIV) epidemic in 1985, case reports of GBS associated with HIV infection have been published, mainly during the seroconversion period. Presentation and prognosis may be identical to cases not related to HIV infection, although atypical presentations, including recurrent episodes of GBS, have been reported.

Although antiretroviral therapy reduced the number and severity of neurological complications, HIV-associated GBS is still a rare disease presentation. A higher incidence is reported in African HIV patients [1, 2].

The HIV tropism for the central nervous system (CNS) is established within the first weeks through the migration of infected CD4+ T lymphocytes or infected monocytes, creating a virus reservoir [4]. In GBS, two mechanisms are proposed: 1) direct inflammatory action of HIV on the nerves; 2) an autoimmune process with the development of antibodies against the myelin sheath [1].

Cerebrospinal fluid (CSF) studies in patients with GBS are usually normal earlier in the course of the disease. Later it exhibits an albuminocytologic dissociation with elevated protein and cell counts in the normal range [4]. However, in HIV patients, CSF studies may differ due to a mild lymphocytic pleocytosis [5, 6].

Plasmapheresis and intravenous immunoglobulin (IVIG) demonstrated equal efficacy, but the latter is the current mainstay of GBS treatment because of a more convenient administration [1]. Corticosteroids can be added to the therapeutic regimen, but evidence of benefit is scarce [4, 6]. As more cases related to HIV infection are being reported, the evidence recommends that antiretroviral therapy (ART) should also be initiated early in the course of the disease to improve the outcome [6]. The choice of a regimen with good CNS penetration provides a rapid reduction of CSF HIV RNA levels [2].

In this article, we describe a case of GBS as the initial presentation of HIV infection, with atypical neurological findings and limited response to immunoglobulin treatment initially. The patient started to improve after three weeks of antiretroviral medication.

## Case report

A 52-year-old man was admitted to the emergency room reporting pain, numbness, and weakness of both legs for the previous three days. He mentioned progressive difficulty walking and was not able to flex his feet, particularly the right foot. In clinical examination, he presented weakness in both upper and lower limbs, being slightly more pronounced on the right side, and the deep tendon reflexes were abolished in all limbs. Besides a history of vocal cord palsy during childhood, the patient was healthy.

Regarding the epidemiological context, the patient had spent nine years working in Africa and returned to Portugal 6 years ago. During his stay in Africa, he reported unprotected sexual contact. Despite no recent history of respiratory or gastrointestinal infections, a GBS diagnosis was sought, and a lumbar puncture was performed, which revealed to be normal, without evidence of albuminocytologic dissociation.

Blood tests revealed cytolytic hepatitis, elevated serum γ-glutamyl transpeptidase level, and a positive antibody test for HIV-1 (Table 1). After a positive confirmatory test was obtained, lymphocyte phenotyping revealed a CD4 cell count of 219/mm3 (22.8%), and blood HIV-1 viral load measurement estimated a value of 97,800 copies/mL. Antiretroviral treatment (tenofovir-emtricitabine and dolutegravir) was initiated. The patient received IVIG treatment for five days at a 0.4 g/Kg/day dose with no response. At this stage, the patient had a flaccid tetraparesis, maintaining asymmetry of muscle strength (grade 4/5 on the upper left limb and grade 3/5 on the lower left limb; grade 3/5 on the upper right limb and grade 2/5 on the lower right limb - with Medical Research Council grade). He was unable to feed himself without help and presented urinary retention. Deep tendon reflexes remained abolished, and neuropathic pain medications were introduced, including paroxetine and pregabalin.

**Table 1:** Laboratory investigations

Sample	Parameters evaluated	Value
Blood	Aspartate-aminotransferase (ALT)	190 U/L (3xULN)
	Alanine-aminotransferase (AST)	112 U/L (3 × ULN)
	γ-glutamyl transpeptidase	192 (N < 55 U/L)
	Hemoglobin, blood glucose, uremia, creatinine, ions, alkaline phosphatase, total bilirubin,lactic dehydrogenase (LDH), creatine-phosphokinase, C-reactive protein, immunoglobulins (total IgG, IgM, IgA), coagulation tests	Negative or within normal ranges
	Antinuclear antibodies and Antiganglioside antibodies	Negative
	HIV antigen/antibody (Ag/Ab) test **˗** CD4+ cell count and % **˗** CD4+/CD8+ ratio **˗** HIV viral load **˗** HLA-B*57:01	Positive 219/mL; 22.8% 0.38 97,800 copies/mL Detected
	Hepatitis B virus markers	HBsAb positive, total HBcAb, and HBsAg: negative
	Hepatitis C Virus-Ab	Negative
	Syphilis screening	Negative
	Cytomegalovirus (CMV), Epstein–Barr virus (EBV), herpes simplex virus (HSV) 1/2	IgG Ab positive
Cerebrospinal Fluid (first sample)	Cells	1/mm3
	Proteins	35 mg/dL
	Glucose	71 mg/dL (95 mg/dL blood glucose)
	Oligoclonal IgG bands, testing for JC virus and cryptococcus, Meningitis/Encephalitis Panel	Negative
	HIV viral load	13,200 copies/mL
Cerebrospinal Fluid (a week after)	Cells	1/mm3
	Proteins	68 mg/dL
	Glucose	70 mg/dL (94 mg/dL blood glucose)
	Culture for bacteria and fungi	Negative

Due to the asymmetric presentation, a spinal cord magnetic resonance was requested to exclude a structural cause. The exam had no relevant findings. A lumbar puncture was repeated seven days after hospitalization that revealed a mild protein elevation (68 mg/dL) without increased cell count. Nerve conduction studies confirmed the diagnosis of a sensory-motor demyelinating polyneuropathy.

The second regimen of IVIG was administered for another five days (14 days after clinical onset/admission) associated with dexamethasone 15 mg/day. On the eighteenth day after the onset of symptoms, neurological signs stabilized, and a few days later, they started to improve. After a month of therapy, laboratory reassessment showed an elevation of CD4 lymphocyte counts (460/mm3 representing 31.5%) and a decrease in HIV-RNA to an undetectable level.

The patient was transferred to a rehabilitation centre. He had partially regained muscular strength on discharge, presenting a grade 3/5 on the right lower limb, grade 4−/5 on the left lower limb, and a full recovery of upper limb strength.

Four months after the hospitalization, he attended a medical appointment, walking without any support and only referring to the difficulty in flexing his right foot.

### Interpretation

Central or peripheral neurological complications are common in HIV-infected patients [6]. If HIV is not treated, a constant inflammatory state will damage neurons [6, 7]. In addition to neurotoxins, an autoimmune response is aroused by changes in membrane components, antigenic viral particles, and disruption of B cells. Antibodies against myelin have been detected in HIV patients with AIDP [8] but not in the reported case.

Several cases of GBS, as the first presentation of the disease, can be found in the literature supporting the recommendation that HIV screening should be part of the investigation in healthy individuals with newfound neurological symptoms [9]. A diagnosis of HIV infection was made following this indication.

The case presented here had some atypical features: 1) although the patient had an asymmetric clinical presentation, he fulfilled the two main criteria for GBS diagnosis (progressive motor weakness and areflexia), and the electromyography supported this hypothesis; 2) the first lumbar puncture did not show elevated protein or pleocytosis, and pleocytosis remained not evident in the second lumbar puncture as would be expected in an HIV patient; 3) a late onset of HIV-associated GBS, with a low CD4 count in the initial evaluation.

Concerning the treatment, promptly initiating ART with a regimen of IVIG 0.4 g/kg/day for five days was in line with current scientific evidence [2, 5, 10, 11]. The lack of response in the first week may have been partly due to the progressive phase of the disease. The second regimen of IVIG and corticosteroids was prescribed to slow down the evolution of the disease, even though corticosteroids proved to be of value only in chronic inflammatory demyelinating polyneuropathy [12, 13].

Autonomic dysfunction, namely urinary retention, was verified. Nevertheless, as described in other case reports [11, 14], clinical recovery was noticed with the preconized treatment and after a stabilization period. In this case, only a minimum strength deficit in the right foot remained, and the patient is possibly still in the recovery phase.

Concerning the ART chosen, emtricitabine and dolutegravir both have good CSF penetration. Tenofovir only has a Central Nervous System Penetration-Effectiveness Ranking (CPE) of 1 in 4. Abacavir (score 3/4) plus emtricitabine and dolutegravir could not be introduced, given the presence of HLA-B57*01. The blood tests showed a rapid reduction of HIV RNA levels, increasing about 10% in CD4 percentage.

There are some references in the literature of recurrent GBS episodes in a higher frequency in HIV-infected individuals than in HIV-negative individuals. So, the patient must be advised that in case a new neurological symptom appears, his/her medical assistant should be contacted promptly.

As a final remark in a case of suspected GBS, HIV screening in the initial blood tests should be requested due to ARTs favourable impact on the evolution of this neurological complication.

## Data Availability

Not applicable.

## Abbreviations

AIDP: Acute inflammatory demyelinating polyneuropathy
ART: Antiretroviral therapy
CNS: Central nervous system
CPE: Central nervous system penetration effectiveness ranking
CSF: Cerebrospinal fluid
GBS: Guillain-Barre syndrome
HIV: Human immunodeficiency virus
IVIG: Intravenous immunoglobulin
